# Can thiamine substitution restore cognitive function in alcohol use disorder?

**DOI:** 10.1093/alcalc/agad017

**Published:** 2023-03-18

**Authors:** Stephan Listabarth, Benjamin Vyssoki, Rodrig Marculescu, Andreas Gleiss, Magdalena Groemer, Armin Trojer, Christine Harrer, Sabine Weber, Daniel König

**Affiliations:** Clinical Division of Social Psychiatry, Department of Psychiatry and Psychotherapy, Medical University of Vienna, 1090 Vienna, Austria; Clinical Division of Social Psychiatry, Department of Psychiatry and Psychotherapy, Medical University of Vienna, 1090 Vienna, Austria; Department of Laboratory Medicine, Medical University of Vienna, 1090 Vienna, Austria; Center for Medical Statistics, Informatics and Intelligent Systems, Institute of Clinical Biometrics, Medical University of Vienna, 1090 Vienna, Austria; Clinical Division of Social Psychiatry, Department of Psychiatry and Psychotherapy, Medical University of Vienna, 1090 Vienna, Austria; Clinical Division of Social Psychiatry, Department of Psychiatry and Psychotherapy, Medical University of Vienna, 1090 Vienna, Austria; Clinical Division of Social Psychiatry, Department of Psychiatry and Psychotherapy, Medical University of Vienna, 1090 Vienna, Austria; Clinical Division of Social Psychiatry, Department of Psychiatry and Psychotherapy, Medical University of Vienna, 1090 Vienna, Austria; Clinical Division of Social Psychiatry, Department of Psychiatry and Psychotherapy, Medical University of Vienna, 1090 Vienna, Austria

**Keywords:** alcohol use disorder, thiamine, alcohol dementia, memory, cognitive impairment, Wernicke encephalopathy

## Abstract

**Aims:**

While clinical consequences of thiamine deficiency in alcohol use disorder (AUD) are severe, evidence-based recommendations on dosage, type of administration and duration of thiamine substitution (TS), and its’ target levels remain sparse. This study aimed to compare the effect of two best practice TS regimens on thiamine blood levels (i.e. thiamine pyrophosphate, TPP) and cognitive function.

**Methods:**

In 50 patients undergoing in-patient alcohol-withdrawal treatment, TPP levels were determined at baseline and end of weeks 1, 2 and 8 following administration of oral TS (3 × 100 mg/day for 7 days followed by 1 × 100 mg/day thereafter) either with or without preceding intravenous TS (3 × 100 mg/day for 5 days). An extensive psychiatric assessment was conducted at baseline, including an evaluation of AUD severity and depressive symptoms. Additionally, cognitive function and depressive symptoms were repeatedly evaluated.

**Results:**

Relevant increases (mean increase by 100.2 nmol/l [CI 76.5–123.8], *P* < 0.001) in peripheral blood TPP levels were observed in all patients at the end of weeks 1 and 2. Furthermore, no relevant difference between the intravenous and the oral group was found (average difference between increases: 2.3 nmol/l, *P* = 0.912). Importantly, an association between the ‘extent of the response’ to TS and the performance in a memory task was revealed in secondary analyses.

**Conclusion:**

TS was associated with improving cognitive function in patients with AUD, independently of the substitution regime. Thus, in clinical practice, oral TS might be a sufficient but obligatory medication to prevent cognitive decline in AUD in the absence of Wernicke–Korsakoff Syndrome.

## Teaser

**Short Summary:** Thiamine deficiency is a frequent condition in patients with alcohol use disorder and associated with cognitive decline and dementia. Two thiamine substitution regimens were evaluated and increases in thiamine levels were associated with pronounced improvements of cognitive function. Interestingly, oral and intravenous substitution seemed to be equally effective.

## Introduction

Alcohol use disorder (AUD) is associated with the development of multiple psychiatric (e.g. affective and anxiety disorders) and somatic disorders (e.g. liver diseases, pancreatitis, and malignancies). Importantly, malnutrition and severe vitamin deficiencies can occur. Among these, vitamin B1 deficiency (thiamine deficiency; TD) is known to be the most relevant and one of the most frequent conditions—with an estimated prevalence of 15–80% in patients with AUD (Morgan 1982; Li et al. 2008). The most severe manifestation of TD is Wernicke encephalopathy (WE)—a potentially life-threatening condition described to manifest with a typical triad of symptoms: eye movement disorder, ataxia, and confusion (Sinha et al. 2019). However, the complete triad is postulated to be present in only ~10% of patients, which may result in numerous undiagnosed cases (Donnino et al. 2007).

Although the pathomechanism underlying the development of WE is not fully understood, the presence of TD is widely accepted as the deciding factor for the emergence of this neuropsychiatric condition (Martin et al. 2003; Chandrakumar et al. 2019; So et al. 2019): the brain almost exclusively relies on glucose utilization and the bioactive derivate of thiamine, thiamine pyrophosphate (TPP), is an essential co-factor for multiple enzymes crucial for glucose metabolism (Martin et al. 2003). This is hypothesized to explain the particularly deleterious effect of TD. Suggested consequences of TD in the brain include the accumulation of neurotoxic compounds (reactive oxygen species) and impaired functioning of the blood–brain-barrier (Martin et al. 2003).

Adequate thiamine substitution (TS) is the only known treatment of WE and may lead to complete remission. Furthermore, TS prevents the progression to Wernicke–Korsakoff Syndrome (WKS)—the chronic and irreversible form of WE. Accordingly, decreased thiamine monophosphate and TPP levels in acute cases of WE and, congruently, clinical improvement with increasing TPP levels after administration of thiamine were reported (Tallaksen et al. 1993a). Additionally, multiple (international) guidelines recommend TS in all cases of symptomatic or even suspected WE but also favour prophylactic TS in all patients with signs and symptoms of AUD (Thomson et al. 2002; Caputo et al. 2019). However, a recent analysis of more than 7500 patients treated in an emergency department in the United States revealed that only as few as 2.2% of all patients with an alcohol-related diagnosis (any of the diagnoses of the ICD-10 F10 category) and 17.8% of all patients with the explicit diagnosis of AUD received administration of thiamine (Peck et al. 2021).

Importantly, while TS is of utmost importance in the prevention and treatment of WE and WKS, also a causal link between TD and mild cognitive impairment (MCI) is suggested: data from autopsy studies have shown reduced enzyme activities in specific brain regions a long time prior to the onset of WE, potentially explaining early impairment of cognitive functioning (Lavoie and Butterworth 1995; Martin et al. 2003). Accordingly, improvement of cognitive function after intramuscular TS has been reported (Ambrose et al. 2001) and an association between whole blood TPP levels and memory was reported in a cross-sectional study for patients diagnosed with AUD (Pitel et al. 2011). Furthermore, TS has been suggested as a possible preventative agent in alcohol-related dementia and Alzheimer’s dementia (Gibson et al. 2016; Chou et al. 2019; Listabarth et al. 2020; Fessel 2021). Considering these findings, it seems reasonable to evaluate an even broader indication of TS as a preventative and/or therapeutic measure in patients with AUD and MCI or even maybe for all patients with AUD–as suggested by Agabio and Leggio (2021).

Despite representing a curative treatment for a potentially life-threatening condition, knowledge of the recommended dosage, duration, and the form of application are still limited (Day et al. 2013; Pruckner et al. 2019). Moreover, until today, neither threshold values of blood thiamine (and phosphate esters) levels marking TD nor a recommended range of blood levels for patients receiving TS due to neuropsychiatric conditions associated with TD (i.e. WE) or for patients receiving TS as a prophylactic treatment in AUD have been agreed on (Whitfield et al. 2018). In summary, evidence regarding TS is scarce, despite the well-known and severe consequences of TD.

Thus, the aims of the current study were: (i) to quantify the effect of TS on TPP levels in patients with AUD; (ii) to analyse if there is a significant difference in the response of TPP levels in patients receiving oral TS compared with those with intravenous administration; (iii) to analyse if the change of TPP levels is associated with a change in cognitive performance; and (iv) to analyse if baseline TPP levels are a relevant predictor for the change in cognitive performance.

## Methods

### Data

#### Study population

This study was conducted at the Department for Psychiatry and Psychotherapy of the Medical University of Vienna (Austria) between November 2019 and April 2021. Patients admitted for in-patient alcohol withdrawal treatment were screened consecutively for inclusion and exclusion criteria upon consent to participate in this study. To be eligible for enrolment, patients had to meet diagnostic criteria of alcohol dependence according to ICD-10 (code F10.2), be between 18 and 65 years of age, and have adequate language skills. Exclusion criteria comprised the presence of a condition accompanied by impaired cognitive functioning, such as any type of dementia, other neurodegenerative diseases, or specific psychiatric conditions, such as severe depression with or without psychotic symptoms, schizophrenia spectrum, and other psychotic disorders (ICD-10 F2.X). Additionally, patients with ongoing 5-fluorouracil treatment, a medical history of bariatric surgery, or previously diagnosed eating disorders (i.e. anorexia nervosa or bulimia nervosa) were excluded from the study, as all these circumstances could potentially interfere with thiamine metabolism. Furthermore, patients who had received any form of thiamine substitution (TS) within the last 4 weeks were also excluded from this study. Considering that human storage capacity for thiamine lasts for ~18 days (Onishi et al. 2018), the latter exclusion criterium was established to ensure comparability of baseline levels. Following the standard of care, patients were required to remain abstinent throughout the whole in-patient stay and withdrawal symptoms were treated with oxazepam in a stepwise reduction. Abstinence was maintained by all patients throughout the study period and tested for with multiple breath-tests throughout the day.

According to best clinical practice, a standardized TS regimen was advised to all patients: 5 days of intravenous 100-mg thiamine chloride hydrochloride in 100-ml 0.9% sodium chloride infusion 3 times per day, followed by 7 days of 3 times daily 100 mg orally administered thiamine administration, followed by 100 mg orally administered daily. In the case of any contraindication for intravenous administration or according to patient preference, an oral treatment regimen was applied with 7 days of 3 times daily 100-mg thiamine administration, followed by 100 mg daily. In both groups, the oral substitution with 100-mg thiamine per day was recommended as a permanent substitution beyond the duration of this study.

This study was approved by the Ethics committee of the Medical University of Vienna (EK-No.: 1809/2019) and was conducted in accordance with the Declaration of Helsinki.

#### Assessments

**Figure 1:**
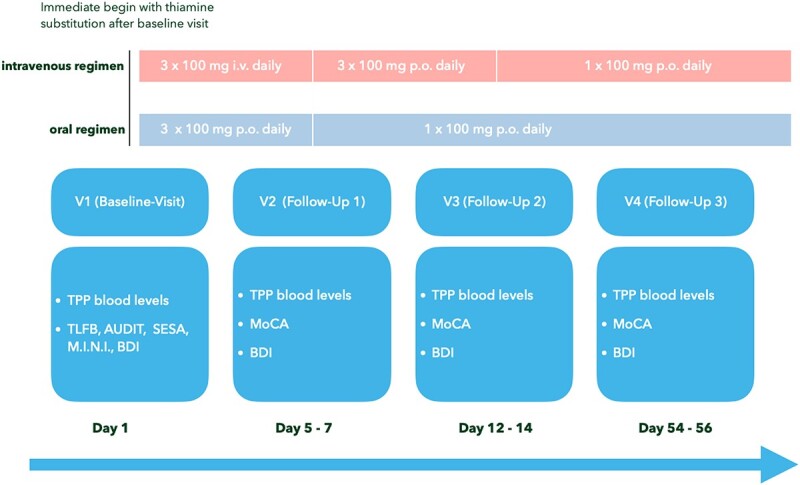
Study design; visit (V), thiamine pyrophosphate (TPP), Montreal Cognitive Assessment (MoCA), Beck Depression Inventory (BDI).

Assessments of TPP levels were conducted at baseline (i.e. visit 1, prior to initiation of TS), at visit 2 (day 5–7), visit 3 (day 12–14), and at visit 4 (day 54–56). Thiamine doses (independent of the route of administration) were administered at 8:00 a.m., 12:00 a.m., and 4:00 p.m. during stages with multiple administrations and at 8:00 a.m. only at stages with once daily administration. Blood tests were always conducted directly before the first thiamine administration of the day (or prior to the first thiamine administration after initiation of inpatient treatment) and before breakfast (i.e. before 8:00 a.m.). Additionally, an extensive psychiatric assessment was conducted at visit 1, including an evaluation of AUD severity and depressive symptoms. At visits 2, 3, and 4, alongside the repeatedly measurement of the severity of depressive symptoms, also the cognitive function was assessed. An overview of the full study design is depicted in Fig. 1.

Considering thiamine’s and its esters sensitivity to light exposure and temperature changes, adequate pre-analytical handling was ensured by processing the specimens cold and light-protected. Subsequently, TPP concentrations in whole blood were determined with the ‘Vitamin B1 Whole Blood - HPLC’ Kit from ChromSystems (Gräfelfing/Munich, Germany) according to the manufacturer’s instructions. Analyses were performed on a Dionex UltiMate 3000 HPLC system equipped with an RF 2000 fluorescence detector (Thermo Fisher Scientific, Waltham, MA). Biomaterial was processed and stored according to standard operating procedures by the Biobank of the Medical University of Vienna in an ISO 9001:2015-certified environment (Haslacher et al. 2018).

Cognitive function was evaluated using the ‘Montreal Cognitive Assessment’ (MoCA, German Version 7). To enable valid retesting within a short time interval for the longitudinal observation of cognitive functioning while limiting the measurement of learned answers, the three parallel versions (7.1–7.3) of the MoCA were used. Previous literature has repeatedly validated the MoCA, supported its use in patients with AUD (Pelletier et al. 2018; Hagen et al. 2019) and revealed a superior sensitivity regarding the detection of MCI when compared with similar tests (i.e. Mini-Mental State Examination) (Nasreddine et al. 2005). Alcohol consumption levels were retrieved using the ‘Timeline-Followback-Method’ (Sobell and Sobell 1992), which allows for retrospective determination of the daily average alcohol consumption in gram alcohol per day using a calendar to reach the highest accuracy possible and is widely used to quantify substance use for research purposes. Additionally, the ‘Severity Scale of Alcohol Dependence’ (SESA) was administered (John et al. 2003). Furthermore, the ‘Beck Depression Inventory’ (BDI-II) was used to evaluate the presence and/or the severity of depressive and anxiety symptoms, respectively.

### Statistical analysis

According to previous studies (Deblon 2007), a standard deviation (SD) of 20 ug/l at each timepoint of measurement was expected, resulting in an estimated SD of 14 ug/l for the arithmetic mean of the TPP levels from visit 2 and visit 3. Thus, with a Pearson correlation between visit 1 and this mean of 0.4 or higher, the SD of change V1 to mean of V2 and V3 is below 20 μg/l. Based on this SD, a one-sample *t*-test at two-sided significance level of 0.05 can detect a clinically relevant change of 10 μg/l with 90% power if 44 patients are included.

Categorical variables are described as counts and percentages and compared between the type of TS using Fisher’s Exact test; continuous variables as mean and SD in case of approximate normal distribution (group comparison using *t*-test), and as median and quartiles (group comparison using Wilcoxon’s rank-sum test) otherwise.

As prespecified in the study protocol, TPP levels of visits 2 and 3 are averaged to obtain more stable estimates of what is assumed to quantify the ‘TPP level after TS’. Due to drop-outs between visits 1 and 3, the change from baseline to the average of visits 2 and 3 was only available for 45 of the 50 included patients. As there were missing values only at visit 3 for 3 of these patients, their TPP levels were imputed from visit 2 levels for a sensitivity analysis, which thus included 48 patients. TPP levels are compared between ‘baseline’ and the ‘average of visits 2 and 3’ using a paired *t*-test after graphically confirming an approximate normal distribution for the intra-individual differences. The mean change is reported with 95% confidence interval. An analysis of covariance (ANCOVA) model was used to compare the change of TPP levels from baseline to the average of visits 2 and 3 between two modes of treatment application while adjusting for baseline TPP levels, age, and average daily alcohol consumption. The mean group difference is reported with 95% confidence interval.

The association between the change in TPP levels from baseline to the average of visits 2 and 3 and the change in MoCA score is quantified using Spearman’s correlation coefficient. A similar exploratory examination was performed for each of the seven subdomains of the MoCA. The most prominent of these associations is investigated using an ANCOVA model with the change of MoCA subdomain score between visits 2 and 3 as the dependent variable and a quadratic function of change in TPP levels from baseline to the average of visit 2 and 3, MoCA subdomain score at visit 2, average daily alcohol consumption, and BDI-II at visit 2 as independent variables (the last two representing the most important adjustment variables; age and gender were removed). *P*-values of this model must be interpreted with care due to the exploratory character of this post-hoc analysis. *R*-square values are reported to quantify the proportion of variation in the dependent variable explained by the set of independent variables; the partial *R*-square quantifies the increase in *R*-square by adding an independent variable to an existing set of variables.

The potential of baseline TPP levels to predict the change of MoCA score from visit 2 to 3 is investigated in a linear regression model with adjustment for age and average daily alcohol consumption.

All statistical analyses were performed using SAS 9.4 (SAS Institute Inc., Cary, NC 2016). *P*-values below 0.05 are regarded to indicate statistical significance. No correction for testing multiple secondary hypotheses was performed due to their exploratory character.

## Results

### Description of the study population

**Figure 2:**
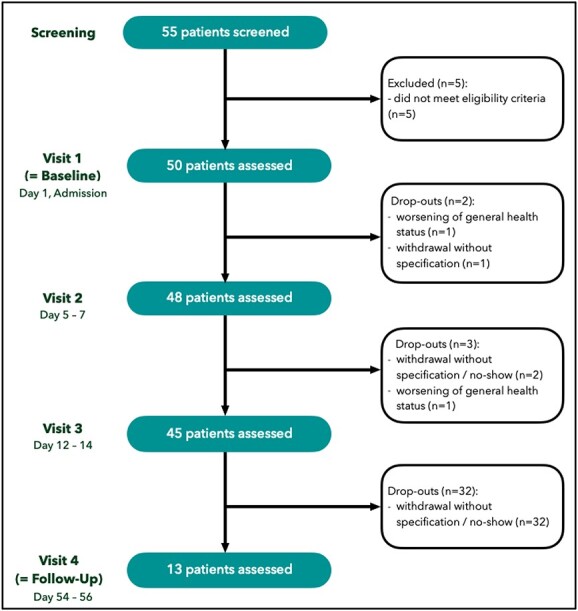
Participants flow chart.

Of the 55 patients screened for inclusion/exclusion criteria, 50 were included in the study. The final study population consisted of 27 male (54.0%) and 23 female (46.0%) patients; the overall mean age was 47.9 years of age (SD 8.5 years). The study population’s median AUDIT score of 32.0 (Interquartile range, IQR, 26.5–34.0) at baseline reflects a severe alcohol use disorder. Further characteristics of the study population are displayed in Table 1. Participants’ flow and drop-out events are depicted in Fig. 2. None of the patients had reported the presence of delirium tremens prior to admission or had developed symptoms of delirium tremens during withdrawal treatment.

**Table 1:** Baseline characteristics of study population.

	*n* = 50
**Sex**	**Frequency (%)**
Female	23 (46.00)
Male	27 (54.00)
**Age**	**Mean (± Std Dev)**
	47.9 (± 8.5)
**Education level**	**Frequency (%)**
Compulsory School	12 (24.00)
Secondary Education	29 (58.00)
Tertiary Education	9 (18.00)
**Alcohol consumption**	**Median (IQR)**
Daily alcohol intake (gram)	154.0 (107.0–200.0)
Heavy drinking days	30.0 (23.0–30.0)
**Psychometric scores**	**Median (IQR)**
AUDIT	32.0 (26.5–34.0)
SESA	60.9 (47.0–70.7)
BDI	20.0 (13.5–29.5)
**Thiamine administration**	**Frequency (%)**
Intravenous	39 (78.00)
Oral	10 (20.00)
Different treatment regimen	1 (2.00)

Daily alcohol intake in gram, average daily alcohol intake over the last 30 days in gram pure alcohol ingested; heavy drinking days, number of heavy drinking days within the last 30 days defined as more than 50-g pure alcohol per day for men and more than 40 g for women; AUDIT, Alcohol Use Disorder Identifcation Test.

### Effect of thiamine substitution on TPP levels

**Figure 3:**
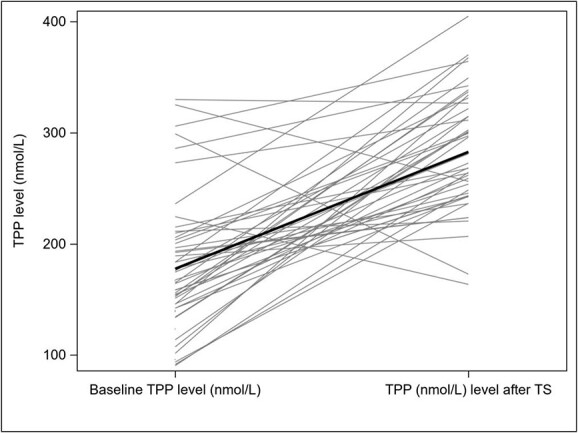
Change of TPP levels independent of administration type (grey: raw data for 45 patients; black: mean values).

With an average increase of 100.2 nmol/l (CI 76.5–123.8), a significant increase (*P* < 0.001) in thiamine pyrophosphate (TPP) levels was revealed between baseline TPP levels and the mean of TPP levels of Visit 2 (i.e. day 5–7) and Visit 3 (i.e. day 12–14). The course of TPP levels is depicted in Fig. 3.

When the TPP levels of Follow-up visit 2 were used to impute the unknown mean of Follow-up visits 2 and 3 for the three patients who dropped out between these two visits, the results remained significant and hardly changed (104.4 nmol/l, CI 80.9–127.9, *P* < 0.001).

**Figure 4:**
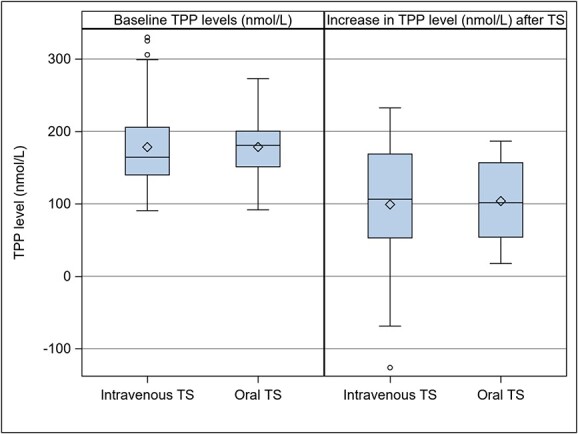
Baseline TPP levels and changes from baseline to after TS by administration type (37 patients with intravenous TS, 10 patients with oral TS).

Interestingly, no relevant difference was revealed when comparing the change of TPP levels of the intravenous and the oral administration group (see Fig. 4 and Table 2). When adjusted for age and average daily alcohol consumption, the difference between these two groups was only marginal, with an on average 2.3 nmol/l higher increase of TTP levels in the intravenous group compared with the oral group (CI −38.0/+42.7 nmol/l, *P* = 0.912).

**Table 2:** Baseline TPP levels and changes from baseline to after TS by administration type (37 patients with intravenous TS, 10 patients with oral TS).

Thiamine administration	Variable	Median	Lower Quartile	Upper Quartile
Intravenous (*n* = 37)	Baseline	164.3	139.9	205.7
	(V2 + V3)/2	270.6	243.6	331.4
	∆TPP	106.3	52.9	168.8
Oral (*n* = 10)	Baseline	180.7	151.2	200.3
	(V2 + V3)/2	289.1	248.5	310.1
	∆TPP	101.6	54.0	156.8

(V2 + V3)/2, arithmetic mean of TPP levels of visit 2 and visit 3; ∆TPP, difference between baseline TPP levels and arithmetic mean of TPP levels of visit 2 and visit 3.

### TPP alteration and cognitive functioning

When evaluating the association between the change of TPP levels induced by TS (change between baseline TPP and mean of TPP levels at visits 2 and 3) and the change of cognitive function (i.e. overall MoCA score) between the first and the second assessment (i.e. visit 2 and 3), no relevant correlation was found (Spearman’s correlation coefficient = 0.16; *P* = 0.31). However, when examining the relevant subdomains of MoCA, a prominent association of ‘delayed recall’ and TPP changes was revealed: in an exploratory regression analysis of the dependence of the change of MoCA subdomain of delayed recall (visit 3 minus visit 2) on the change of TPP levels (as defined above), a statistically significant quadratic effect was revealed [*P* = 0.041 not corrected for multiple testing; partial *R*^2^ = 0.11, when added to daily average alcohol consumption, depressive symptoms (BDI-II total score) and the score of the MoCA subdomain of delayed recall of visit 2]. Removal of two extreme residual outliers yielded even stronger evidence (*P* = 0.007; partial *R*^2^ = 0.23).

### Baseline TPP levels as predictor for cognitive functioning

A regression model representing the potential impact of baseline TPP levels on the later development of cognitive functioning (change of overall MoCA score between visits 2 and 3) adjusted for age and daily average alcohol consumption did not reach statistical significance (*P* = 0.925, *R*^2^ = 0.04). Furthermore, when adding the TPP levels of Visit 2 as a potential predicting variable for the change of cognitive functioning, the model’s explanatory power did not improve notably (*R*^2^ = 0.10).

## Discussion

To our knowledge, this is the first longitudinal study comparing the effect of oral and intravenous TS on TPP blood levels in patients with AUD undergoing inpatient withdrawal treatment. A significant increase in TPP levels after the initiation of TS was observed in our study population. Importantly, a significant association with mean changes in TPP levels and relevant subdomains of the MoCA was revealed. Interestingly, the observed increases in TPP did not differ between the group with intravenous TS and the group with oral TS—after pre-interventional differences between the two groups were ruled out by a comparison of means at baseline (see Table S1 ‘Baseline characteristics of study population stratified for type of TS’ in the supplementals).

Regarding the cognitive function of patients with AUD but without WE, the analysed data revealed a significant positive correlation between the change in TPP levels after TS and delayed memory: the more pronounced the increase in TPP levels, the greater the improvement in memory function. Importantly, the revealed effect of the increase of thiamine levels on cognitive function was independent of the expected improvement of cognition as a secondary consequence of the withdrawal treatment itself (e.g. the consequence of prolonged abstinence). Furthermore, while a potentially detrimental effect of withdrawal treatment with benzodiazepines (i.e. oxazepam) on cognitive function is to be assumed, this factor applied to the entirety of the study population and, thus, accentuates the revealed increase in cognitive function associated with an increase in thiamine levels. However, to minimize distortion due to either alcohol intoxication at the time of admission or interference through withdrawal treatment with benzodiazepines, the primary assessment of the cognitive function in this study was conducted not before visit 2.

The fact that thiamine status might be particularly affecting the cognitive dimension of memory is in line with previous research in animal models (Langlais et al. 1992; Ciccia and Langlais 2000; Vedder et al. 2015) as well as in humans (Baker and Frank 1976; Thomson et al. 1983; Ambrose et al. 2001; Pitel et al. 2011; Coulbault et al. 2019; Dingwall et al. 2022). However, some of these studies conducted in humans used cross-sectional approaches to investigate the associations between altered thiamine metabolism and cognitive deficits (Pitel et al. 2011; Coulbault et al. 2019). The clinical evidence on the extent of reversibility of these deficits through TS is, however, limited: in a preliminary randomized, double-blind, multidose study by Ambrose et al. (2001) in which AUD patients received a daily dosage of either 5, 20, 50, 100, or 200 mg of intramuscular thiamine, a dose depending effect of thiamine on cognitive functioning, specifically on working memory, was reported. However, this finding could not be replicated in a consecutively conducted study (Dingwall et al. 2022) by the same research group. Further data are limited to studies conducted ~40 years ago (Baker and Frank 1976; Thomson et al. 1983). In these latter studies, however, employed methods are no longer recommended, and also the observed clinical outcome focused on the presence of neurological symptoms, whereas cognitive function had not explicitly been evaluated.

In summary, the results of this study add to existing evidence, suggesting that TS can improve cognitive functioning in patients with AUD. The present study’s findings are even more remarkable, as none of the included patients exhibited symptoms of WE or WKS, which suggests that also patients in less advanced stages of cognitive impairment (compared with those seen in WKS patients) benefit from TS. In further consequence, this has considerable implications for clinical practice as this suggests a much broader indication for TS, including those found to have elevated alcohol consumption (e.g. in screening examinations at general practitioners). Especially considering the dramatic increase in alcohol-related dementia (Gupta and Warner 2008) and reports on the crucial contribution of alcohol consumption to disease progression in any type of dementia (Schwarzinger et al. 2018; Nogueira et al. 2021), this aspect seems highly relevant.

It seems reasonable to assume that these results can be generally translated to patients with AUD as the analysed study collective shows similar characteristics concerning average alcohol consumption, age, and severity of AUD (i.e. AUDIT-score) when compared with those of other studies comprised of patients diagnosed with AUD (Coulbault et al. 2019; Pitel et al. 2011). Also, baseline TPP levels of our study population (184.71 ± 61.1 nmol/l) correspond to reported baseline levels of other cohorts of patients with AUD (Tallaksen et al. 1992; Mancinelli et al. 2003; Deblon 2007; Dingwall et al. 2022). In the study of Tallaksen et al. (1992), for example, a mean TPP level of 149 ± 64 nmol/l for male and 130 ± 30 nmol/l for female patients with AUD was reported. However, it is crucial to consider the limited comparability of thiamine determination using HPLC, as there is no standardization for thiamine HPLC determination and, thus, different HPLC methods have been applied. Furthermore, and probably even more limiting, pre-analytic procedures also widely differ, as concluded in a review by Lynch and Young (2000).

While the significant increase of TPP blood levels after TS had been anticipated, the lack of a relevant difference between oral and intravenous administration observed was surprising. Previous literature has suggested intravenous administration to be superior to oral administration. Tallaksen et al. (1993b) reported a distinct difference in bioavailability between intravenous and oral administration, with only 5.3% relative availability for the latter. Importantly, this pharmacokinetic analysis of a single oral vs. single intravenous thiamine administration of 50 mg was conducted in a population of healthy individuals. Also, multiple guidelines recommend intravenous TS for patients with diagnosed AUD undergoing withdrawal treatment (Thomson et al. 2002; Caputo et al. 2019). Contrarily to these previous assumptions, data from the present study indicate an equal efficacy of oral and intravenous TS—at least regarding blood TPP levels.

The question remains to what extent inferences on brain thiamine metabolism can be drawn from peripheral thiamine status (i.e. blood TPP levels). While evidence on direct measurement of brain thiamine status in humans is lacking, data from animal studies suggest that oral administration of thiamine increases brain thiamine levels and, in further consequence, prevents brain lesions (Suzuki et al. 2017). Previous literature suggests that a high concentration gradient between plasma and the central nervous system—for example, supraphysiological thiamine levels accomplished by high-dose intravenous TS—may be essential for rapid thiamine uptake into the brain (Thomson 2000). Thus, one may assume that peak—rather than average—levels of TPP in the blood are relevant for restoring adequate brain thiamine levels. Subsequently, in TS, it should be aimed for the highest possible bioavailability. The latter might be especially important when considering the relatively short half-life of unphosphorylated thiamine (~100 min Tallaksen et al. 1993b). Thus, multiple administrations per day might be advantageous for rapid brain thiamine restoration. Following this approach, some studies have investigated the effect of thiamine derivatives with a significantly higher bioavailability compared with regular thiamine, such as the S-acyl derivative benfotiamine or the thiamine disulfide derivative sulbutiamine: whereas higher blood levels compared with regular TS were reported, no difference in brain thiamine levels was found (Volvert et al. 2008; Xie et al. 2014). Furthermore, the study collective analysed in the present study may, in agreement with the absence of overt WE specific symptoms, represent a selection of patients in whom the impairment of the thiamine metabolism had not yet progressed to the extent that could only be compensated by intravenous TS. Therefore, the current data do not allow forgoing intravenous TS, particularly in patients with more severe manifestations of TD. This is even more relevant in light of recent findings suggesting a genetic predisposition for WE (O’Brien et al. 2022).

Nevertheless, the currently insufficient knowledge of the targeted therapeutic threshold, the temporal interactions between TS and recovery of cognition, and further factors (e.g. utilization of thiamine esters in patients with alcohol-related liver disease, the presence of additional nutritional deficiencies such as magnesium deficiency) potentially interfering with thiamine metabolism complicates research efforts concerning this matter (Martin et al. 2003; Dingwall et al. 2022). In the present study, all deficiencies identified using routine blood tests were supplemented. The above mentioned aspects could be responsible for obfuscating the above-described expected benefits of intravenous high-dose TS (derived from preclinical and pharmacokinetic studies) in clinical studies (see also Nakamura et al. 2018). However, as these questions could only partially be addressed in the present study, some questions regarding TS’s specific mode of administration remain open. Thus, future research should aim to gain more insights into the underlying mechanisms explaining the connection between peripheral and brain thiamine metabolism, ideally by developing direct but noninvasive brain thiamine determination methods. Other aspects that would be important to consider in future prospective studies are longer follow-up periods in order to learn more about the long-term effects, as well as an attempt to objectify the degree of malnutrition (e.g. by body-mass-index, evaluation of the individual diet).

## Limitations

The following limitations must be considered when interpreting the results of this study. Evidently, a study design including a control group of AUD patients with treatment as usual (i.e. excluding any type of TS) would have been preferable. However, withholding TS to patients undergoing alcohol detoxification would not have been feasible due to ethical reasons. Additionally, only a few patients were recruited in the group with oral TS. This is, however, a result of the chosen study design, according to which primarily intravenous TS was recommended to the patients and only in the presence of any contraindications or due to patients’ preference, oral TS was administered.

## Conclusions

The present study indicates that intravenous and oral substitution of thiamine is equally efficient in raising peripheral TPP levels in patients with AUD but no overt neurological condition (i.e. WKS). Thus, it seems reasonable to consider, at least for patients without signs of WE, oral TS as equally sufficient compared with parenteral TS. Additionally, an association between the response to TS and change in memory function was revealed. The latter finding highlights the crucial role of thiamine deficiency in cognitive deterioration due to excessive alcohol consumption and underscores the importance of TS as a preventative measure in these patients. Future research should investigate to what extent peripheral (i.e. blood) levels of thiamine esters correlate with thiamine uptake at the actual site of action, which is the brain.

## Supplementary Material

supplementary_agad017

## Data Availability

The data underlying this article cannot be shared publicly due to the privacy of individuals that participated in the study. The data will be shared on reasonable request to the corresponding author.
