# Supplementary material for: Can thiamine substitution restore cognitive function in alcohol use disorder?
Source: Alcohol Alcohol. 2023 Mar 18;58(3):315–23. doi: 10.1093/alcalc/agad017 (PMC10168713; doi:10.1093/alcalc/agad017)
Supplement: supplementary_agad017 [file supplementary_agad017.pdf]

	Intravenous TS	Oral TS	Intrav. vs. oral TS	Different TS regimen
**n =**	39	10	p-value	1
**Gender**	**Frequency (%)**	**Frequency (%)**		**Frequency (%)**
Female	18 (46.15)	4 (40.00)	1.000[a]	1 (100.00)
Male	21 (53.85)	6 (60.00)		0 (0.00)
**Age**	**Mean (± Std Dev)**	**Mean (± Std Dev)**		**Mean (± Std Dev)**
	47.7 (± 8.9)	48.8 (± 7.8)	0.728[b]	48.0 (± NA)
**Education_level**	**Frequency (%)**	**Frequency (%)**		**Frequency (%)**
Compulsory School	8 (20.51)	4 (40.00)	0.346[a]	0 (0.00)
Secondary Education	24 (61.54)	4 (40.00)		1 (100.00)
Tertiary Education	7 (17.95)	2 (20.00)		0 (0.00)
**Alcohol consumption**	**Median (IQR)**	**Median (IQR)**		**Median (IQR)**
Daily alcohol intake (gram)	165.0 (112.0 – 200.0)	152.0 (107.0 – 195.0)	0.980[c]	96.0 (NA)
Heavy drinking days	30.0 (23.0 - 30.0)	29.5 (23.0 - 30.0)	0.966 [c]	30.0 (NA)
**Psychometric Scores**	**Median (IQR)**	**Median (IQR)**		**Median (IQR)**
AUDIT	31.0 (25.0 - 34.0)	32.5 (32.0 - 34.0)	0.660 [c]	34.0 (NA)
SESA	61.4 (46.4 - 70.6)	59.8 (55.3 - 73.7)	0.766 [c]	55.4 (NA)
BDI	20.0 (14.0 - 30.0)	20.0 (11.0 - 27.0)	0.856 [c]	20.0 (NA)
**TPP level in nmol/L**	**Median (IQR)**	**Median (IQR)**		**Median (IQR)**
	164.3 (139.9 - 205.7)	180.7 (151.2 - 200.3)	0.730 [c]	142.2 (NA)

Table S1: Baseline characteristics of study population stratified for type of TS. (Std Dev, Standard Deviation; IQR, Interquartile range; Daily alcohol intake in gram, Average daily alcohol intake over the last 30 days in gram pure alcohol ingested; Heavy drinking days, Number of heavy drinking days within the last 30 days defined as more than 50 gram pure alcohol per day for men and more than 40 gram for women; AUDIT, Alcohol Use Disorder Identifcation Test; SESA, Severity Scale of Alcohol Dependence; BDI – Beck Depression Inventory – II;)

[a] Fisher's Exact Test

[b] T-test

[c] Wilcoxon rank-sum test
